# Rescue Therapy With Factor VII for Refractory Cardiac Surgical Bleeding: A Propensity-Score-Matched Study

**DOI:** 10.1093/icvts/ivaf185

**Published:** 2025-08-12

**Authors:** Victor M Neira, Christian D Neira, Kara Matheson, Matthias Scheffler, Renata Morton, Heather E Mingo, Edgar G Chedrawy, Hashem Aliter

**Affiliations:** Department of Anesthesia Perioperative Medicine and Pain Management, Dalhousie University, Halifax, Nova Scotia, B3H 2Y9, Canada; Department of Anesthesia Perioperative Medicine and Pain Management, Dalhousie University, Halifax, Nova Scotia, B3H 2Y9, Canada; Centre for Clinical Research, Nova Scotia Health, Research Methods Unit, Dalhousie University, Halifax, Nova Scotia, B3H 1V7, Canada; Department of Anesthesia Perioperative Medicine and Pain Management, Dalhousie University, Halifax, Nova Scotia, B3H 2Y9, Canada; Division of Cardiac Surgery, Department of Surgery, Dalhousie University, Halifax, NS, 1796 Summer Street, Halifax, Nova Scotia, B3H 3A6, Canada; Division of Cardiac Surgery, Department of Surgery, Dalhousie University, Halifax, NS, 1796 Summer Street, Halifax, Nova Scotia, B3H 3A6, Canada; Division of Cardiac Surgery, Department of Surgery, Dalhousie University, Halifax, NS, 1796 Summer Street, Halifax, Nova Scotia, B3H 3A6, Canada; Division of Cardiac Surgery, Department of Surgery, Dalhousie University, Halifax, NS, 1796 Summer Street, Halifax, Nova Scotia, B3H 3A6, Canada

**Keywords:** Factor VII, bleeding, cardiac surgery, refractory

## Abstract

**Objectives:**

To compare effectiveness and safety of rescue therapy approach with recombinant Factor VII activated (rFVIIa) for refractory bleeding in cardiac surgery compared with a propensity-score-matched control group at a single academic institution.

**Methods:**

In total, 8860 adult patients had cardiac surgery with cardiopulmonary bypass between 2009 and 2019. Ninety-seven patients (1.1%) received rFVIIa; 81 (83.5%) of rFVIIa cases were propensity score matched 1:1 with controls using pre- and intraoperative variables. Effectiveness was assessed with coagulation tests, chest tube drainage, and reoperation for bleeding. Safety was assessed with morbi-mortality.

**Results:**

The median dose of rFVIIa was 55.6 μg/kg (IQR, 37.4-80.0 μg/kg). The first dose after CPB was given at a Median time of 176 min (IQR, 131-232 min). Postoperative INR was lower in the rFVIIa group (Median, 0.8; IQR, 0.7-0.9) versus control (Median, 1.4; IQR 1.3-1.6; *P* <.0001). Other coagulation tests, chest tube drainage, and reoperation for bleeding were no different. Mortality and thrombo-embolism were higher in the rFVIIa—OR, 3.17 (95% CI, 1.41-7.14; *P* = .0054) and OR, 10.50 (95% CI, 1.64-117.5; *P* = .0196). Stroke (OR, 1.82; 95% CI, 0.51-6.48; *P* = .35) and renal failure (OR, 1.31, 95% CI, 0.69-2.48, *P* = .41) were not statistically different. RFVIIa group received 4.4 (95% CI, 3.28-5.91, *P* = .0001) and 1.97 (95% CI, 1.18-3.30; *P* = .02) times more blood products volume intra- and postoperatively.

**Conclusions:**

Rescue therapy with rFVIIa seems to effectively control bleeding. However, we observed an association with increased mortality, thromboembolism, and transfusion. We did not find rFVIIa association with risk of stroke or renal failure.

## Graphical abstract

**Figure:**
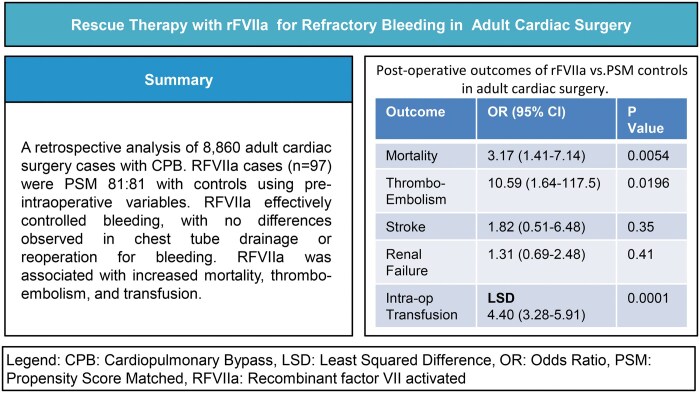

## INTRODUCTION

The off-label use of recombinant Factor VII activated (rFVIIa) for the treatment of refractory bleeding in cardiac surgery remains controversial.^1^^,^^2^ The management of bleeding and coagulopathy after complex and emergency cardiac surgeries is challenging, associated with transfusion, shock, re-exploration for bleeding, morbidity, and mortality.^3^ Despite blood management and hemostasis guidelines in cardiac surgery, around 50% of elective cardiac surgery patients receive blood products.^3–5^ Transfusion algorithms incorporating point of care coagulation tests targeting haemostatic treatments seem to decrease bleeding and transfusion.^5^^,^^6^ However, a small percentage of patients remain refractory to treatments and may require recombinant rFVIIa to control bleeding.^3^^,^^7^

A recent review highlighted the contradictory information regarding current recommendations for the use of rFVIIa in cardiac surgery and its supporting evidence.^1^ The 2021 clinical practice guidelines on patient blood management in cardiac surgery states a level IIB recommendation for low dose (20-40 µg/kg) rFVIIa: “may be considered for intractable non-surgical bleeding unresponsive to routine haemostatic therapy after cardiac procedures using cardiopulmonary bypass (CPB).”^5^ The current European Guidelines (2024) for blood management in cardiac surgery provides similar recommendations, reserving its use as a “last resource only in patients with uncontrollable bleeding refractory to conventional interventions.”^8^ This “rescue therapy” approach is based on historical data.^7^^,^^9–13^ More recent studies (single institution case series and propensity score) suggest that early administration of rFVIIa at a low (20-40 µg/kg) or at a very-low dose (10-20 µg/kg) are effective controlling the bleeding without increasing mortality or morbidity.^14–16^ Our institutional guidelines restricted the use of rFVIIa for cardiac surgery patients with non-surgical bleeding refractory to conventional management.

The primary objective of this study was to investigate effectiveness of rFVIIa for the treatment of refractory bleeding in cardiac surgery assessed by surgeons’ documentation of acceptable bleeding control, reoperation for bleeding, and chest tube drainage at 12, 24, and 48 h. The secondary objective was to investigate safety: mortality, stroke, renal failure, embolic complications, blood transfusion requirements, and cell saver use compared with a propensity-score matched group.

## METHODS

Institutional Ethics Board approval (File N. 1026267, February 8, 2021) was obtained, and patient consent was waived for this retrospective observational single-centre propensity score-matched study. It followed the STROBE guidelines for reporting observational studies.^17^ This study data storage is consistent with the WMA Declaration of Taipei. The institutional cardiac surgery database was used to identify adult patients undergoing cardiac surgery with CPB between 2009 and 2019. Patients who received rFVIIa perioperatively in cardiac surgery with CPB (cases) identified in the blood bank database were 1:1 propensity-score matched with ones who did not (controls), using pre- and intraoperative variables.

The inclusion criterion for the study group was the administration of rFVIIa in cardiac surgery with CPB intra- or postoperatively. Patients who received rFVIIa based on a medical condition, for cardiac surgery without CBP, or non-cardiac surgery were excluded. Data were retrieved from institutional databases and the electronic medical records. Variables of interest in the rFVIIa group were the total dose, dose/kg, timing of administration after separation from CPB, and number of doses.

All patients received general anaesthesia, anticoagulation with heparin (400 UI/kg for an ACT> 480 sec), reversal with protamine 1:1-1.5, and tranexamic acid bolus of 10 mg/kg and infusion of 5 mg/kg/h intraoperatively. Transfusion of blood products was at the discretion of the team, guided by point of care tests: blood gases, haemoglobin, thromboelastography (2012-2014), rotational thrombo-elastometry (2015-current) according to contemporary guidelines.^4^ Our blood transfusion triggers for packed red blood cells (PRBC) is haemoglobin (Hb) ≤ 70 g/l, fresh frozen plasma (FFP) or prothrombin concentrate: EXTEM-CT > 100 s, fibrinogen: FIBTEM-A10 < 10 mm, and platelets (PLT) a PLT count ≤ 100 000/µL or EXTEM-A10 < 40 mm.^3^^,^^6^

Effectiveness was investigated comparing effective bleeding control as documented in surgeons’ note, reoperation for bleeding, chest tube drainage at 12, 24, and 48 h, and pre- and post-rFVIIa administration PRBC transfusion.

Safety was investigated comparing in hospital mortality (within 30 days), stroke (neurologic deficits documented in the chart or by neuroimaging), renal failure (requirement of renal replacement therapy or dialysis), and thrombo-embolic complications. Other variables of interest included preoperative creatinine, Euroscore, LVAD, ECMO, redo sternotomy, pre- and postoperative coagulation tests, haemoglobin, blood products transfused intra and up to 48 h postoperatively, total intraoperative fluids administered, estimated bleeding and cell saver use.

All blood products at our institution are component fractions according to the Canadian Blood Services.^18^ For clarity, authors decided to describe blood products in volume and not in units. Intra-operative bleeding is not routinely measured in our institution.

### Statistical analysis

Propensity Score Matching was completed through the institutional cardiac surgery database. Patients who received rFVIIa in cardiac surgery with CPB were the study group. All other cases who underwent cardiac surgery with CPB were included in the propensity score analysis to identify the control group. Twenty-five baseline pre- and intraoperative covariates for propensity score analysis was chosen *a-priori* based on clinical significance (**Electronic Supplementary Material S1**). Authors specified *a priori* that having the year of admissions within a range of ± 2 years, which all matched pairs met, was acceptable to control for potential changes in practice over time. A logistic regression was used to generate a propensity score, and the probability of rFVIIa treatment assignment was conditional on observed baseline covariates. Greedy nearest neighbour matching without replacement was then used to select treated subjects and a matched control subject if the absolute difference in their propensity scores was within the pre-specified maximal calliper distance of 0.01.^19–21^ The authors proposed a standardized difference value of > 0.1 indicating an imbalance in the baseline covariates.^21^

A sensitivity analysis was performed to look at the impact of any missing data in the original dataset. Missing values were replaced with the mean covariate value for continuous variables and a dichotomous variable was included in the model to indicate if the value was missing or not.

The standardized difference for each covariate was used to quantify the magnitude of the difference between baseline characteristics of the treated and untreated groups. Covariate imbalance was also examined by testing covariates for statistical differences between the 2 groups. Univariable logistic regression models were then run to estimate the treatment effect on the outcomes; mortality, stroke, renal failure, and thrombo-embolism. Exploratory multivariable models were run which incorporating covariates included in the propensity score model to explore associations between baseline variables and outcome as well to eliminate residual imbalance.^20^

Comparisons between rFVIIa group and the matched control group were performed using Wilcoxon signed-rank test for ordinal and continuous variables, and McNemars’ test for categorical variables.

Comparison between rFVIIa group and the matched control group for the transfusion outcomes were performed using negative binomial regression modelling. A *P*-value of < .05 was used to indicate statistical significance. SAS STAT 14.3 software, version 9.4 was used for all statistical analyses.

## RESULTS

### RFVIIa group

**Figure 1.**
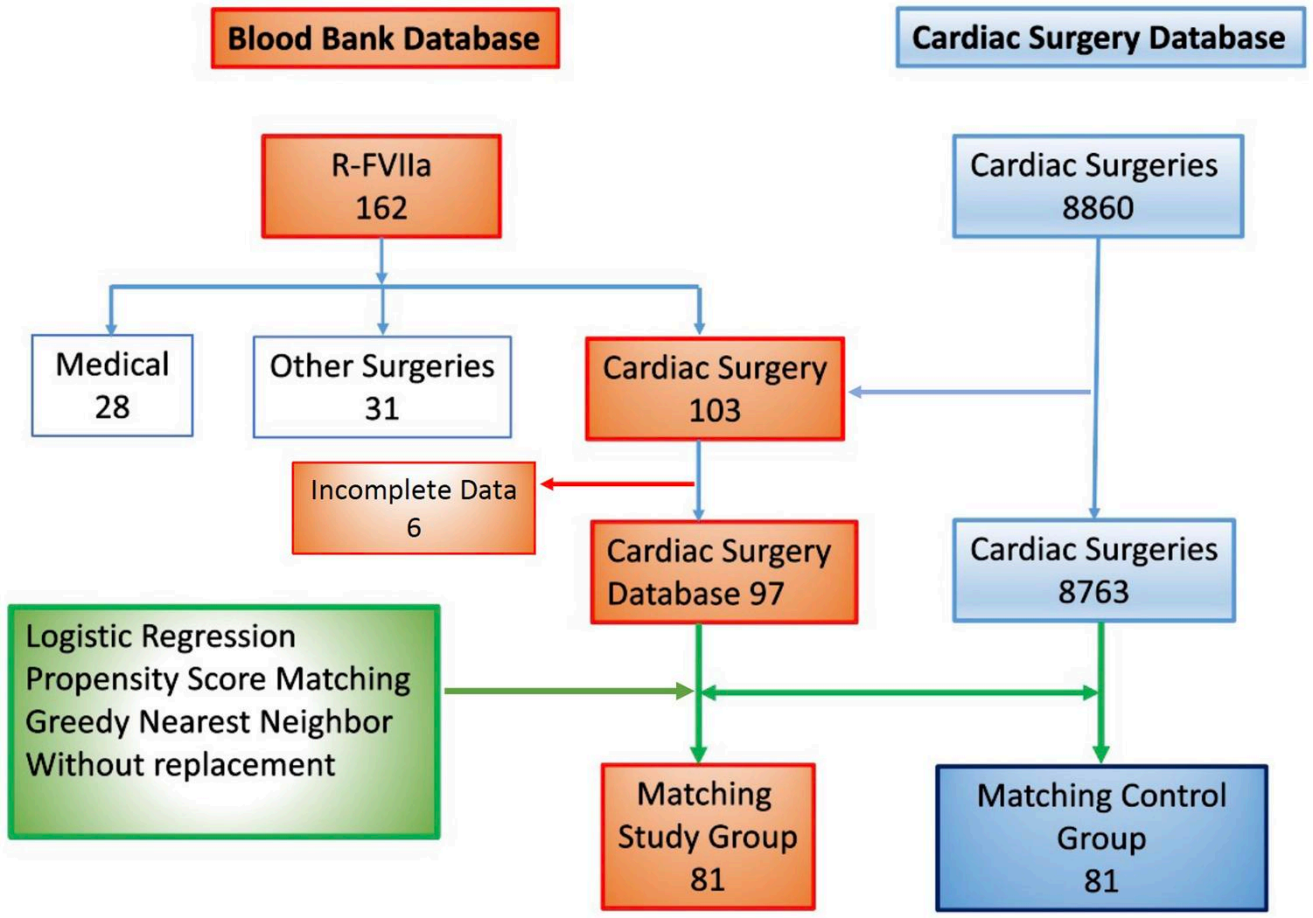
Flowchart of Cases Included in the Study. R-FVIIa: recombinant Factor VII activated

One-hundred-sixty-two patients (*n* = 162) received rFVIIa for any reason from January 1, 2009, to December 31, 2019, from which 103 were cardiac surgery patients with CBP (**Figure 1**). The Cardiac Surgery Database had 8860 cardiac surgeries with CPB, and 97 patients (1.1%) who received rFVIIa peri-operatively were identified. Six cases were excluded due to missing data. The median total dose of rFVIIa was 55.6 µg/kg with high variation—interquartile range (IQR), 42.62 µg/kg, and a mean first dose of 42 µg/kg (SD ±20.5 µg/kg). Sixty-four patients received one dose of rFVIIa, 33 patients received more than 1 dose. The median time for the first dose of rFVIIa after separation from CPB was 176 min (IQR, 131-232 min). RFVIIa was administered in the operating room for the index operation in 75% (*n* = 58), in the CVICU in 12.9% (*n* = 10) and 11.6% (*n* = 9) in the reoperation for bleeding. The 30-day mortality (36%, *n* = 35) patients received significantly higher doses of rFVIIa compared to survivors (Median, 70.80 μg/kg; IQR, 45.53-114.0 vs Median, 50.85; IQR, 30.30-67.80; *P* = .0026, respectively).

**Electronic Supplementary Material S2** depicts the pre- and intraoperative variables for the 97 rFVIIa patients in the Cardiac Surgery database, and the 8763 patients in the Cardiac Surgery Database that did not receive rFVIIa. The pre-match rFVIIa and non-rFVIIa population showed significant differences in 20 clinically relevant variables. Prior to matching, 21 (84%) of 25 variables had a standardized difference of > 0.1 compared to 6 (29%) after matching, being all ≤ 0.20, without statistical differences (**Electronic Supplementary Material S1**). Eighty-one rFVIIa cases (83.5%) were matched 1:1 with controls, which resulted in substantial reductions in the imbalance of covariates based on standardized difference and significance tests while still maintaining optimal calliper distances (**Table 1**; **Electronic Supplementary Material S2**). Preoperative haemoglobin, INR, APTT, platelets, creatinine, REDO sternotomy, LVAD, ECMO, and intraoperative circulatory arrest were not different between groups (**Electronic Supplementary Material S3**). In contrast, the EuroSCORE was significantly different between groups (rFVIIa Median, 24.84; IQR, 10.59-43.3 and Control Median, 14.98; IQR, 4.20-29.37; *P* = .0018).

**Table 1.** Recombinant FVII in Cardiac Surgery Post-Matching Standardized Differences

		Post-matching	
Factor	Matched control (n = 81)	Matched rFVIIa (n = 81)	*Standardized difference post-match*
**Demographics**			
Age, y, median, IQR	63, 55.0-72.0	64.0, 50.0-73.0	0.061
Male sex, n (%)	56 (69.1%)	56 (69.1%)	0.000
Year of the procedure (within 2 y), median, IQR	9.4, 7.8-11.9	9.8, 7.4-10.6	0.201
**Comorbidities**			
Cardiogenic shock, n (%)	3 (3.7%)	4 (4.9%)	0.061
PVD, n (%)	34 (42.0%)	29 (35.8%)	0.127
Cerebrovascular disease, n (%)	16 (19.8%)	13 (16.0%)	0.097
CKD/Dialysis, n (%)	10 (12.3%)	15 (18.5%)	0.171
Diabetes, n (%)	14 (17.3%)	16 (19.6%)	0.064
PHT, n (%)	6 (7.4%)	7 (8.6%)	0.045
COPD, n (%)	15 (18.5%)	14 (17.3%)	0.032
Infectious endocarditis, n (%)	3 (3.7%)	3 (3.7%)	0.000
CHF, n (%)	23 (28.4%)	22 (27.2%)	0.028
**Preoperative medications**			
Ace inhibitor/RA, n (%)	46 (56.8%)	42 (51.9%)	0.099
ASA/Clopidogrel, n (%)	70 (86.4%)	64 (79.0%)	0.128
Heparin, n (%)	19 (23.5%)	20 (24.7%)	0.029
Inotropes, n (%)	6 (7.4%)	6 (7.4%)	0.000
**Intraoperative**			
Lowest core temperature, °C, median IQR	30.1 (25.0, 32.2)	30.1 (24.9, 32.1)	0.005
Clamp time, median (IQR)	127.0 (87.0-195.0)	133.0 (77.0-185.0)	0.011
CPB time, median (IQR)	237.0 (146.0-281.0)	222.0 (152.0-295.0)	0.079
**Type of surgery**			
CABG, n (%)	16 (19.8%)	11 (13.6%)	0.166
Valve(s), n (%)	30 (37.0%)	30 (37.0%)	1.000a
Aortic root, n (%)	13 (16.0%)	19 (23.5%)	0.1797
Aortic root dissection, n (%)	8 (9.9%)	9 (11.1%)	0.7630
Transplant/LVAD, n (%)	14 (17.3%)	12 (14.8%)	0.6171
**Surgical priority**			
Elective, n (%)	19 (23.5%)	22 (27.2%)	0.085
Inhouse, n (%)	46 (56.8%)	41 (50.6%)	0.124
Emergent/urgent, n (%)	16 (19.8%)	18 (22.2%)	0.061

ASA, aspirin; CHF, congestive heart failure; CKD, chronic kidney disease; COPD, chronic obstructive pulmonary disease; CPB, cardio-pulmonary bypass; IQR, interquartile rank; LVAD, left ventricular assist device; PHT, pulmonary hypertension; PVD, peripheral vascular disease; rFVIIa, recombinant Factor VII activated.

Effectiveness: There were no differences between groups in the surgeons’ documentation of effective bleeding control, chest tube drainage at 12, 24, 48 h, ICU APTT, ICU PLTs, or reoperation for bleeding (including cases who received rFVIIa during the index surgery *n* = 56). There were significant differences in the ICU INR, being lower in the rFVIIa group (Median, 0.8; IQR, 0.7-0.9) versus control (Median, 1.4; IQR, 1.3-1.6; *P* < .0001) (**Electronic Supplementary Material S3**). The number of intraoperative PRBC units administered was higher before rFVII administration (Mean 4.21 SD ± 2.89) versus after (Mean 1.85 SD ±1.83, *P* < .0001).

### Safety outcomes

Mortality rate was higher in the rFVIIa group compared to controls—30.8% (*n* = 25) vs 12.3% (*n* = 10); (OR, 3.17; 95% CI, 1.41-7.14; *P* = .0054). Thrombo-embolic events were also higher in the rFVIIa group 11.1% versus 1.23% in the control group (OR, 10.59; 95% CI, 1.64-117.5; *P* = .0196). Stroke rates were 8.6% for the rFVIIa group versus 4.9% for the control group (OR, 1.82; 95% CI, 0.51-6.48; *P* = .35). Renal failure rates were 39.5% for the rFVIIa group versus 33.3% for the control group (OR, 1.31; 95% CI, 0.69-2.48; *P* = 0.41) (**Table 2**).

**Table 2.** Comparison of Safety Outcomes Between rFVIIa Group and Control

Outcome	OR (95% CI)	*P*-value
Mortality	3.17 (1.41-7.14)	.0054^a^
Thrombo-embolism	10.59 (1.64-117.5)	.0196^b^
Stroke	1.82 (0.51-6.48)	.35^a^
Renal failure	1.31 (0.69-2.48)	.41^a^

a Covariate Wald test.

b McNemar’s matched pair test.

**Figure 2.**
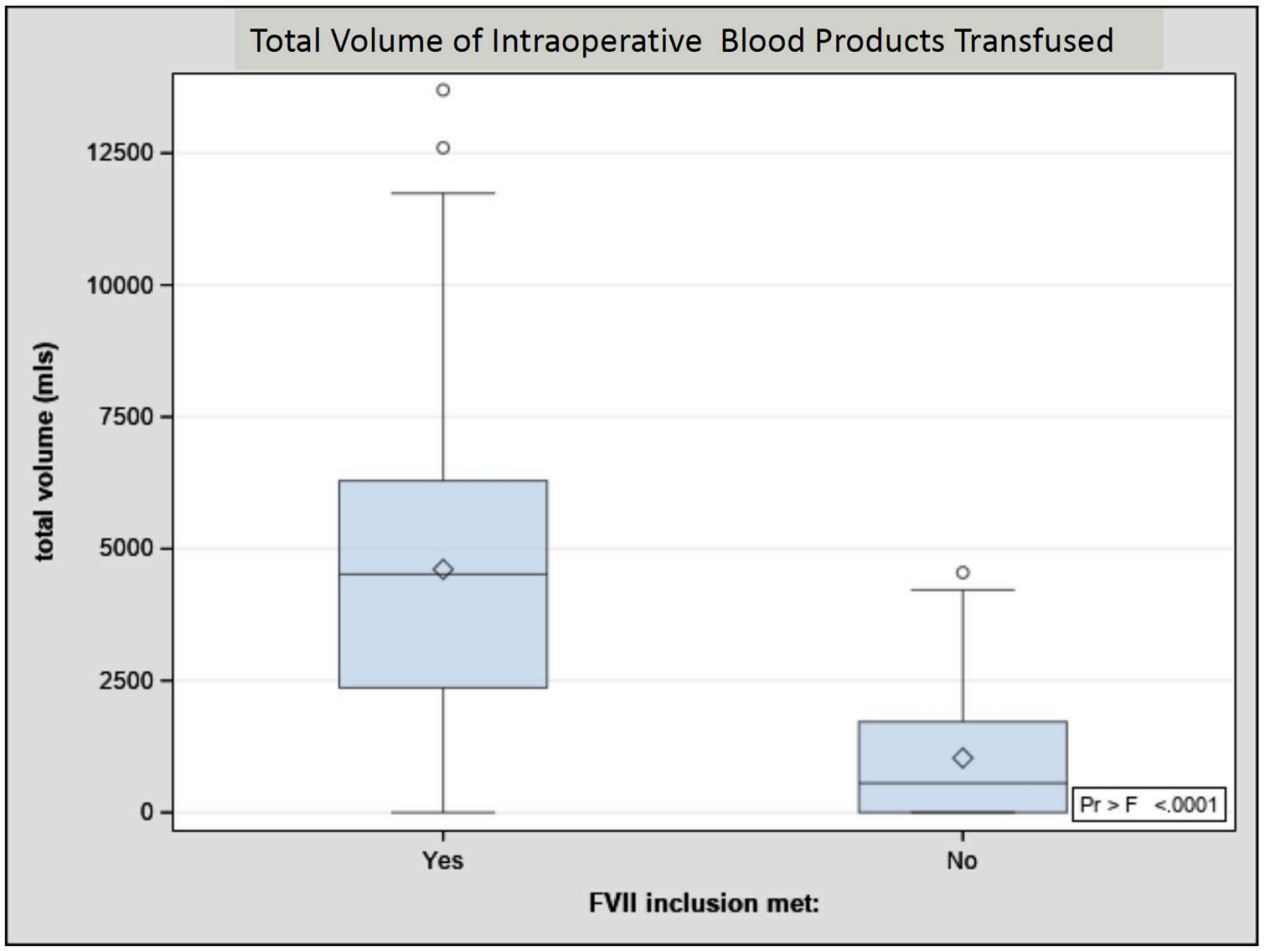
Comparison of Total Volume of Blood Products Transfused Intraoperatively to Cardiac Surgery Patients Receiving Recombinant FVII Activated (rFVIIa) Versus a Propensity Score Matching Group

The rVIIa group received 4.4 (95% CI, 3.282-5.91; *P* = .0001) times more blood products volume intraoperatively than the propensity matched control, and nearly 2 times more in the postoperative period (OR, 1.97; 95% CI, 1.18-3.30; *P* = .02). The total mean blood products transfused volume was 4608 mL (SD ± 3117 mL) vs 1033 mL (SD ± 1215 mL), *P* < .0001(**Figure 2**). PRBC were the most frequently transfused product. Detailed comparisons are shown in the **Electronic Supplementary Material S4**. Within the rFVIIa group, mortality cases received more PRBC transfusion (Median, 2095 mL; IQR, 970-3202 mL) compared with the survivor group (Median, 1250; IQR 585-2203 mL; *P* = .015). The total intraoperative fluid administration was higher in the rFVIIa group (Median, 5881 mL; IQR 3677-8385 mL) versus the control group (Median, 1800 mL; IQR 1000-2982 mL; *P* < .0001).

There were no differences between groups in the use of fibrinogen or prothrombin concentrate complexes (**Electronic Supplementary Material S5**). A high standard deviation was found in both groups for both factors. The use of cell saver was also higher in the rFVIIa group (*n* = 41, 51.2%) versus the control (*n* = 23; 28.7%; *P* = .006). The time from CPB separation to the end of surgery was longer in the rFVIIa group (M = 265 min, SD ± 123 min) vs (M = 117 min; SD ± 68 min; *P* = .0001). The time from rFVIIa administration to end of surgery varied widely (Mean: 110 min, SD ± 94 min).

## DISCUSSION

The off-label use of rFVIIa as a rescue therapy for refractory bleeding in cardiac surgery was infrequently employed in our institution, with only 1.1% of our patients receiving it. Our results suggest that rFVIIa is effective in controlling bleeding, since there were no differences in the intraoperative bleeding control between groups when rFVIIa was given in the index operation. The INR was lower postoperatively, and there were no differences in chest tube drainage or re-operations for bleeding. The first dose of rFVIIa was administered close to 3 h post-CPB separation, following intense haemostatic management. However, the rescue approach was associated with triple the risk of mortality, 10 times higher risk of thromboembolic complications, 4 times more transfusion of blood products, over double the time from separation from CPB to the end of surgery, and 2-fold use of cell-saver compared to the matched control group. Although the dose administered was relatively high, it was consistent with contemporary guidelines.^3^ The observed association of rFVIIa use with increased mortality cannot be interpreted as causality. Rather, the authors hypothesize that late administration could have delayed effective bleeding control, resulting in massive transfusion and shock, which independently contributed to morbi-mortality.

Our results are aligned with previous rescue therapy studies which also showed infrequent use with similarly high mortality results.^7^^,^^13^ While our total dose tended to be lower than previous rescue therapy studies, it still exceeded the current recommended dose (20-40 μg/kg).^3^^,^^7^^,^^13^ Our mortality cases received higher doses of rFVIIa than the survivors (70 μg/kg vs 50 μg/kg), a finding not previously reported.^7^^,^^13^

The stroke rate in our cohort was 8.6%, which is lower than previously reported (15% and 17%).^7^^,^^13^ We observed high rates of renal failure without differences between groups, consistent with a previous study, although another one found increased risk in the rFVIIa group.^7,13^ Similar high transfusion volumes have been reported in previous rescue therapy studies (approximately 2200 mL), with varying results.^7^^,^^13^^,^^22^

In contrast, a multicentre propensity-score-matched report for type A aortic dissection repair with 120 matched cases reported that 15% of cases received rFVIIa at a median dose of 5 mg (approximately 62 µg/kg for an 80 kg patient).^22^ That study found a non-significant difference in in-hospital mortality, stroke, and renal replacement therapy.^22^

Rescue therapy approach outcomes contrast with those of early treatment with low (20-40 μg/kg) or very low-dose (<20 μg/kg) of rFVIIa. Overall, very low-dose studies administer rFVIIa to a higher percentage of cases (5%-15%) and repeat the dose according to the response.^16^^,^^23–26^ Several of those publications are retrospective observational studies from the same center.^23–26^ The decision to administer rFVIIa of those studies occurred “at the discretion” of the surgical team, without objective measurement of blood lost or point of care coagulation tests. Two of those studies used the Society of Thoracic Surgeon’s morbidity-mortality score for propensity score matching, however several confounders were significantly different compared to the study (rFVIIa) group.^23^,^25^ Mortality rates (11%) were not different between groups, as well as stroke, renal failure, and hospital length of stay.^23^^,^^25^^,^^27^

Another observational single center propensity-score matching study using very-low dose of rFVIIa used a combined approach of clinical assessment of hemostasis, with real-time laboratory testing, and point of care coagulation tests.^16^ Fourteen percent of cases received rFVIIa and had low mortality rates and other adverse outcomes with no statistical differences.^16^ For many of the propensity score matching studies on the low-dose approach; however, the matching process was very heterogenous, or was not properly disclosed, adding uncertainty to the quality of evidence.^16^^,^^23^^,^^25^

Our institutional guidelines are now considering the use of rFVIIa at a very low dose as a second-line therapy rather than last resort. The 2024 EACTS/EACTAIC Guidelines on patient blood management in adult cardiac surgery, accordingly, advise extreme caution with rFVIIa. Properly designed multicentric RCTs are warranted to address safety concerns, however, ethical concerns are a challenge.^8^ A strength of our study which may inform future study designs, is our finding that late administration of rFVIIa and a dose > 40 µg/kg may increase the risk of morbi-mortality.

Our study has limitations inherent to retrospective single-centre designs, including increased risk of biases and potential for incomplete information. Matching was not based on transfusion rates, as transfusion was predefined as an outcome due to our institutional guidelines reserving rFVIIa as a last-resort therapy. The matching included 25 clinically relevant variables. However, there were persistent standardized difference in 6 variables (year of surgery, peripheral vascular disease, renal disease, aspirin, CABG, and in house) between 0.1 and 0.2, which may introduce residual confounding. However, statistical analysis of the matching covariates was not significant. The higher Euroscore in the rFVIIa group also highlights concerns about the matching process, as some factors (ie, “critical preoperative state”) were not captured in our database matching. The long inclusion period is another limitation, as changes in patients’ population, advances in surgical and blood management practices could affect the results. Interpretation of the results may be impacted based on these limitations.

In conclusion, rescue therapy with rFVIIa was associated with increased mortality, thrombo-embolism, and transfusion requirements as compared with a propensity-matched control group. However, rFVIIa association with stroke or renal failure was not observed.

## Supplementary Material

ivaf185_Supplementary_Data

## Data Availability

Data were accessed from the QE-II HSC, and managed by the Research Methods Unit. Data will be shared on request to the corresponding author with permission of the above-mentioned institutions.
