# Supplementary material for: Rescue Therapy With Factor VII for Refractory Cardiac Surgical Bleeding: A Propensity-Score-Matched Study
Source: Interdiscip Cardiovasc Thorac Surg. 2025 Aug 12;40(8):ivaf185. doi: 10.1093/icvts/ivaf185 (PMC12377900; doi:10.1093/icvts/ivaf185)
Supplement: ivaf185_Supplementary_Data [file ivaf185_supplementary_data.zip › ESM 2 Pre-Post-Matching P val.docx]

**ESM 2.** Comparison of Pre and Intraoperative Factors Before and After Propensity-Score Matching.

|  | **Pre-Matching** | | |  | **Post- Matching** |  |
| --- | --- | --- | --- | --- | --- | --- |
| **Factor** | **Non-rFVIIa Population**  **(n= 8763)** | **rFVIIa Population**  **(n= 97)** | ***P value*** | **Matched Control (n=81)** | **Matched rFVIIa (n=81)** | ***P value*** |
| **Demographics** |  |  |  |  |  |  |
| Age, y, median, IQR | 67.0, 60.0-74.0 | 64.0, 50.0-73.0 | **0.0123^1^** | 63, 55.0-72.0 | 64.0, 50.0-73.0 | 0.7759^1^ |
| Male sex, n (%) | 6549 (74.8%) | 64 (66.0%) | **0.0482^2^** | 56 (69.1%) | 56 (69.1%) | 1.000^2^ |
| Year of the Procedure (within 2y), median, IQR | 8.3 (5.8, 10.8) | 9.9 (7.4, 10.7) | **0.0021^1^** | 9.4, 7.8-11.9 | 9.8, 7.4-10.6 | 0.2240^1^ |
| **Comorbidities** |  |  |  |  |  |  |
| Cardiogenic shock, n (%) | 141 (1.6%) | 7 (7.2%) | **<.0001^2^** | 3 (3.7%) | 4 (4.9%) | 0.6547^2^ |
| PVD, n (%) | 1432 (16.3%) | 32 (33.0%) | **<.0001^2^** | 34 (42.0%) | 29 (35.8%) | 0.4233^2^ |
| Cerebrovascular disease, n (%) | 1247 (14.2%) | 16 (16.5%) | 0.5258^2^ | 16 (19.8%) | 13 (16.0%) | 0.5127^2^ |
| CKD/Dialysis, n (%) | 506 (5.8%) | 17 (17.5%) | **<.0001^2^** | 10 (12.3%) | 15 (18.5%) | 0.2253^2^ |
| Diabetes, n (%) | 3247 (37.1%) | 17 (17.5%) | **<.0001^2^** | 14 (17.3%) | 16 (19.6%) | 0.6831^2^ |
| PHT: n (%) | 430 (4.9%) | 12 (12.4%) | **0.0008^2^** | 6 (7.4%) | 7 (8.6%) | 0.7630^2^ |
| COPD, n (%) | 1122 (12.8%) | 15 (15.5%) | 0.4360^2^ | 15 (18.5%) | 14 (17.3%) | 0.8273^2^ |
| Infectious endocarditis, n (%) | 247 (2.8%) | 4 (4.1%) | 0.4410^2^ | 3 (3.7%) | 3 (3.7%) | 1.000^2^ |
| CHF, n (%) | 1436 (16.4%) | 26 (26.8%) | **0.0060^2^** | 23 (28.4%) | 22 (27.2%) | 0.8227^2^ |
| **Pre-operative Medications** |  |  |  |  |  |  |
| Ace inhibitor/RA, n (%) | 5424 (61.9%) | 49 (50.5%) | **0.0214^2^** | 46 (56.8%) | 42 (51.9%) | 0.52711^2^ |
| ASA /Clopidogrel, n (%) | 7096 (81.0%) | 56 (57.7%) | **<.0001^2^** | 70 (86.4%) | 64 (79.0%) | 0.4249^2^ |
| Heparin, n (%) | 2507 (28.6%) | 23 (23.7%) | 0.2867^2^ | 19 (23.5%) | 20 (24.7%) | 0.8474^2^ |
| Inotropes, n (%) | 205 (2.3%) | 12 (12.4%) | **<.0001^2^** | 6 (7.4%) | 6 (7.4%) | 1.0000^2^ |
| **Intra-operative** |  |  |  |  |  |  |
| Lowest core Temperature, °C, median IQR | 32.7 (31.5-34.3) | 30.4 (27.0-32.2) | **<0.0001^1^** | 30.1 (25.0, 32.2) | 30.1 (24.9, 32.1) | 0.8087^1^ |
| Clamp time, Median (IQR) | 81.0 (62.0-109.0) | 134.0 (70.0-199.5) | **<0.0001^1^** | 127.0 (87.0-195.0) | 133.0 (77.0-185.0) | 0.9754^1^ |
| CPB time, Median (IQR) | 115.0 (90.0-150.0) | 224.0 (152.0-328.0) | **<0.0001^1^** | 237.0 (146.0- 281.0) | 222.0 (152.0- 295.0) | 0.2651^1^ |
| **Type of Surgery** |  |  |  |  |  |  |
| CABG, n (%) | 5183 (59.1%) | 11 (11.3%) | **<0.0001^2^** | 16 (19.8%) | 11 (13.6%) | 0.2752^2^ |
| Valve(s), n (%) | 3180 (36.3%) | 35 (36.1%) | 0.5611^2^ | 30 (37.0%) | 30 (37.0%) | 1.000^2^ |
| Aortic Root, n (%) | 253 (2.9%) | 23 (23.7%) | **<0.0001^2^** | 13 (16.0%) | 19 (23.5%) | 0.1797^2^ |
| Aortic Root Dissection, n (%) | 75 (0.9%) | 13 (13.4%) | **<0.0001^2^** | 8 (9.9%) | 9 (11.1%) | 0.7630^2^ |
| Transplant/ LVAD, n (%) | 72 (0.8%) | 15 (15.5%) | **< 0.0001^2^** | 14 (17.3%) | 12 (14.8%) | 0.6171^2^ |
| **Surgical Priority** |  |  |  |  |  |  |
| Elective, n (%) | 3607 (41.3%) | 23 (25.6%) | 0.0025^2^ | 19 (23.5%) | 22 (27.2%) | 0.5637^2^ |
| Inhouse, n (%) | 4542 (52.0%) | 42 (46.7%) | 0.3128^2^ | 46 (56.8%) | 41 (50.6%) | 0.3532^2^ |
| Emergent/urgent, n (%) | 584 (6.7%) | 25 (27.8%) | **< 0.0001^2^** | 16 (19.8%) | 18 (22.2%) | 0.6374^2^ |

ASA: Aspirin, CHF: Congestive heart failure. CKD: Chronic kidney disease, COPD: Chronic obstructive pulmonary disease, CPB: Cardio-pulmonary bypass, IQR: Interquartile rank, LVAD: Left ventricular assist device, PHT: Pulmonary hypertension, PVD: Peripheral vascular disease,

rFVIIa: recombinant Factor VII activated.

^1^Comparison for continous variables was performed with Wicoxon sign rank test.

^2^ Comparison of categorical variables was performed with McNemars test.
