# Supplementary material for: Rescue Therapy With Factor VII for Refractory Cardiac Surgical Bleeding: A Propensity-Score-Matched Study
Source: Interdiscip Cardiovasc Thorac Surg. 2025 Aug 12;40(8):ivaf185. doi: 10.1093/icvts/ivaf185 (PMC12377900; doi:10.1093/icvts/ivaf185)
Supplement: ivaf185_Supplementary_Data [file ivaf185_supplementary_data.zip › ESM 3 Other Variables 1.docx]

**Electronic Supplementary Material 3**

Comparison of Other Variables of Interest between Matched rFVIIa Groups and Control Group

| Variable | FVII  Median (IQR) | Control  Median (IQR) | p |
| --- | --- | --- | --- |
| Pre-Creatinine | 95 (76.5-123.5) | 95.5 (75.75- 135.5) | 0.5614^1^ |
| EuroScore | 24.84 (10.59-43.3) | 14,98 (4.20-29.37) | 0.0018^1^ |
| Pre-INR | 1 (1-1.3) | 1 (1-1.3) | 0.2215^1^ |
| Pre-APTT (sec) | 32 (29-38.5) | 32 (20-38) | 0.7239^1^ |
| Pre Platelets (*1,000 µL) | 196 (164-141) | 175 (141-240) | 0.1654^1^ |
| 12 h Chest Tube Drainage (mL) | 250 ((100-400) | 250 9150-360) | 0.6445^1^ |
| 24 h Chest Tube Drainage (mL) | 386 (200-600) | 350 (200-550) | 0.6353^1^ |
| 48 Chest Tube Drainage (mL) | 530 (275-840) | 450 (225-700) | 0.4704^1^ |
| Time from CPB off to End of Surgery | 276.5 (226.8-366) | 95 (66-158) | <0.0001^1^ |
| REDO operation | 23 (27.1%) | 27 (34.18%) | 0.1779^2^ |
| Pre-LVAD | 6 (7.8%) | 6 (7.8%) | 1.0000^2^ |
| Pre-ECMO | 1 (1.3%) | 0 | 1.0000^2^ |
| Circulatory Arrest | 22 (28.6%) | 14 (18.2%) | 0.0881^2^ |
| Effective Bleeding Control | 62 (80.5%) | 65 (85.5%) | 0.5316^2^ |
| Reop. For Bleeding^3^ | 12 (8.57%) | 9 (6.43%) | 0.1128^2^ |
| Thrombo-Embolism | 8(11.0%) | 1 (1.3%) | 0.0196^2^ |

Pre-: Preoperative

^1^Sign test.^2^ McNemar’s Test ^3^ Only cases who received rFVIIa in the first operation were included.
