# Supplementary material for: Rescue Therapy With Factor VII for Refractory Cardiac Surgical Bleeding: A Propensity-Score-Matched Study
Source: Interdiscip Cardiovasc Thorac Surg. 2025 Aug 12;40(8):ivaf185. doi: 10.1093/icvts/ivaf185 (PMC12377900; doi:10.1093/icvts/ivaf185)
Supplement: ivaf185_Supplementary_Data [file ivaf185_supplementary_data.zip › ESM5 Comp Coag Factors 2.doc]

**Electronic Supplementary Material 5** Comparison of Coagulation Factor Concentrates between the Matched Control Group and the Factor VII group

|  |  | **Matched Control** | **Matched rFVIIa** | **P-value** |
| --- | --- | --- | --- | --- |
| **Number of Cases** | **Patient received Fibrinogen (g)** |  |  |  |
| **Intra-op initial surgery** | Mean (SD) | 0.99 (2.21) | 1.84 (3.3) |  |
| n (%) | Yes, n (%) | 16 (20.8%) | 26 (33.3%) | 0.0787 |
|  | **Patient received PCC** |  |  |  |
| Dose (IU) | Mean (SD) | 225 (630) | 125 (548.3) |  |
| n (%) | Yes, n (%) | 12 (15.6%) | 8 (10.3%) | 0.3225 |
| **Post-op initial surgery** | **Patient received Fibrinogen (g)** |  |  |  |
| Dose (g) | Mean (SD) | 0.1 (0.63) | 0.31 (1.05) |  |
| N (%) | Yes, n (%) | 2 (2.6%) | 7 (9.0%) | 0.1669 |
|  | **Patient received PCC** |  |  |  |
| Dose IU | Mean (SD) | 18.75 (124.37) | 12.5 (78.55) |  |
| n (%) | Yes, n (%) | 2 (2.6%) | 2 (2.6%) | 1.0000 |

g: grams, IU: International Units, n: Number, PCC: prothrombin concentrate, rFVIIa: Recombinant Factor VII activated, SD: standard deviation
