# Supplementary material for: Rescue Therapy With Factor VII for Refractory Cardiac Surgical Bleeding: A Propensity-Score-Matched Study
Source: Interdiscip Cardiovasc Thorac Surg. 2025 Aug 12;40(8):ivaf185. doi: 10.1093/icvts/ivaf185 (PMC12377900; doi:10.1093/icvts/ivaf185)
Supplement: ivaf185_Supplementary_Data [file ivaf185_supplementary_data.zip › ESM 1 SD Pre and Post Match 3.doc]

**Electronic Supplementary Material Table 1**. Standardized Differences of Pre and Intraoperative factors pre and post-Matching of cardiac surgery cases receiving recombinant factor VII activated for intractable bleeding in cardiac surgery and the matching control.

| **Factor** | **Standardized Difference** | |
| --- | --- | --- |
|  | **Pre-Match** | **Post-Match** |
| **Demographics** |  |  |
| Sex | 0.18 | 0 |
| Age at Surgery | 0.375 | 0.061 |
| Year of Surgery | 0.307 | 0.201 |
| **Comorbidities** |  |  |
| Cardiogenic Shock | 0.257 | 0.061 |
| Congestive Heart Failure | 0.254 | 0.028 |
| Peripheral Vascular Disease | 0.404 | 0.127 |
| Cerebrovascular Disease | 0.038 | 0.097 |
| Infective Endocarditis | 0.03 | 0 |
| Pre-operative Intubation | 0.086 | 0.061 |
| Chronic Obstructive Pulmonary Disease | 0.109 | 0.032 |
| Pulmonary Hypertension | 0.159 | 0.045 |
| Renal Disease | 0.408 | 0.171 |
| Diabetes | 0.442 | 0.064 |
| **Preoperative Medications** |  |  |
| Angiotensin Converting Enzyme Inhibitors | 0.243 | 0.099 |
| Aspirin | 0.53 | 0.128 |
| Clopidogrel | 0.294 | 0.031 |
| Heparin | 0.095 | 0.029 |
| Inotropes | 0.388 | 0 |
| **Intraoperative** |  |  |
| Coronary Artery Bypass Graft | 1.126 | 0.166 |
| **Cardiopulmonary Bypass** |  |  |
| Clamp Time | 0.59 | 0.011 |
| Cardiopulmonary Bypass Time | 1.096 | 0.079 |
| Lowest Core Temperature | 0.902 | 0.005 |
| **Surgical Priority** |  |  |
| Elective | 0.339 | 0.085 |
| Inhouse | 0.107 | 0.124 |
| Emergent/Urgent | 0.582 | 0.061 |
