# Supplementary material for: Rescue Therapy With Factor VII for Refractory Cardiac Surgical Bleeding: A Propensity-Score-Matched Study
Source: Interdiscip Cardiovasc Thorac Surg. 2025 Aug 12;40(8):ivaf185. doi: 10.1093/icvts/ivaf185 (PMC12377900; doi:10.1093/icvts/ivaf185)
Supplement: ivaf185_Supplementary_Data [file ivaf185_supplementary_data.zip › ESM 4 FVII Sec Out. Blood transf. .doc]

| **Intraoperative Blood Products Administered** |  | |
| --- | --- | --- |
|  | **Volume of Blood Products Transfused (mL/kg)** | |
|  | **Control** | **rFVIIa** |
|  | **Mean (SD)** | **Mean (SD)** |
| Weight | 82.75 (18.9) | 83.59 (18.1) |
| PRBC | 5.88 (8.2) | 25.8 (21.4) |
| Apheresis PLT | 0.47 (1.2) | 1.28 (1.8) |
| Pooled PLT | 1.79 (3.3) | 7.36 (5.8) |
| Cryo | 0.03 (0.07) | 0.19 (0.2) |
| FFP | 4.79 (7.1) | 23.41 (20.1) |
| **Total** | 12.96 (15.2) | 58.05 (43.3) |
| LSD (95% CI) | 4.42 (3.26 - 5.98) | |
| **NB *p-value*** | ***<0.0001*** | |
| **Post-Operative Blood Products Administered (48h)** |  | |
| RBC | 2.98 (6.73) | 4.91 (7.01) |
| Apheresis PLT | 0.07 (0.44) | 0.23 (0.75) |
| Pooled PLT | 0.38 (1.25) | 0.63 (0.191) |
| Cryo | 0.01 (0.03) | 0.02 (0.07) |
| Plasma | 2.23 (4.89) | 4.13 (7.51) |
| **Total** | 5.67 (11.68) | 9.91 (14.34) |
| LSD (95% CI) | 1.73(1.00 - 2.99) | |
| ***NB p-value*** | ***0.0467*** | |

**ESM 4.** Comparison of cardiac surgery patients receiving Recombinant Factor VII activated (rFVIIa) and a propensity-score matching group secondary outcomes: Intraoperative Blood products transfusion. Cryo: Cryoprecipitates, FFP: Fresh frozen plasma, LSD: Least squared difference, NB: negative binomial regression model, PRBC: Packed Red Blood Cells, PLT: platelets
